# Peer Interaction in Late-Diagnosed Autistic Adolescent Boys and Girls

**DOI:** 10.3390/jcm15114340

**Published:** 2026-06-04

**Authors:** Miri Ben Shabbat-Seri, Hagit Nagar-Shimoni, Yael Leitner, Annalia Rabinovich-Shefer, Nirit Bauminger-Zviely

**Affiliations:** 1 Faculty of Education, Bar-Ilan University, Ramat-Gan 5290002, Israel; miribss@gmail.com; 2 Marot Autism Center, Child Development Institute, Dana-Dwek Children’s Hospital, Tel Aviv Sourasky (Ichilov) Medical Center, Tel Aviv-Yafo 6423906, Israel; hagitnagar8@gmail.com (H.N.-S.); leitnery@tlvmc.gov.il (Y.L.); 3 Analia Rabinovich Shefer, the Autism Center, Alut, Shamir Medical Center, Zerifin 7030000, Israel; alut@alut.org.il

**Keywords:** autism spectrum disorder, peer interaction, adolescence, biological sex differences, late diagnosis

## Abstract

**Background:** This study examined peer interactions among late-diagnosed autistic adolescents, focusing on biological sex differences. **Methods:** Participants included 61 adolescents aged 12–18 years (31 boys, 30 girls) with an Intelligence Quotient (IQ) > 75, all diagnosed within the past three years. Peer interactions were assessed using the APIOS-A, an adapted version of the preschool observational tool (APIOS). Parents reported on autistic traits via the Social Responsiveness Scale (SRS-2), which measures social communication difficulties; socialization skills through the Adaptive Behavior Assessment System (ABAS-II), which assesses adaptive functioning; and behavioral problems using the Child Behavior Checklist (CBCL), which identifies emotional and behavioral issues. These measures were utilized to compare social profiles across sexes. **Results:** The APIOS-A demonstrated good reliability for identifying socio-communicative difficulties in both boys and girls. Findings indicated that boys and girls showed similar peer interaction challenges, with subtle differences in conversational reciprocity, cognitive flexibility, and humor use. Associations between behavioral characteristics and peer patterns varied by sex, highlighting the importance of tailored approaches. **Conclusions:** These results underscore the need for sex-sensitive assessment tools and tailored interventions for late-diagnosed autistic adolescents. Integrating observational and parental measures may enhance understanding and support their social development.

## 1. Introduction

### 1.1. Peer Interaction in Late-Diagnosed Autistic Adolescent Boys and Girls

Adaptive peer interaction during adolescence is a key component of social–emotional development and well-being, particularly for those diagnosed with autism spectrum disorder (ASD), who face core challenges during social interactions: autistic adolescents may show a desire to connect and form friendships, but they sometimes struggle to understand the social codes required for this [1,2]. Autism is a neurodevelopmental condition characterized by challenges in reciprocal social communication and interaction, alongside restricted and repetitive patterns of behavior, interests, or activities [3]. Research on boys versus girls with autism has revealed biological sex differences in various aspects but has consistently shown autistic girls’ higher quality of social interaction, with stronger social interaction and communication skills than autistic boys [4].

Individuals who receive a late diagnosis of this developmental disorder during adolescence have been comparatively understudied relative to those diagnosed in infancy or early childhood. These late-diagnosed autistic adolescents are usually characterized by less severe autistic symptoms, a higher IQ, and better language abilities than those identified earlier [5], but research on these adolescent boys’ and girls’ peer interactions remains scarce. Specifically, current assessment tools fall short in comprehensively measuring the social-communicative abilities of autistic adolescent boys versus girls during ongoing social interactions. To address the gap in the literature regarding social interaction challenges in autism and considering biological sex-specific characteristics, our study modified the Autism Peer Interaction Observation Scale (APIOS) [6] to explore sex differences in peer interactions among late-diagnosed adolescents with autism.

### 1.2. Peer Interaction in Adolescent Boys and Girls with ASD

Adolescents with autism often experience challenges in interactions with their peers. They may struggle to recognize and understand the emotions of others, which can limit their empathetic responses and affect their social engagement. Additionally, autistic boys and girls are found to exhibit lower levels of visible emotional arousal, suggesting reduced emotional empathy [7]. These challenges include difficulties in initiating and maintaining conversations, sharing emotions, and participating in prosocial behaviors. Difficulties in understanding social norms may further hinder their ability to engage in context-appropriate prosocial behaviors, such as sharing, helping others, or resolving conflicts [1,2,8].

Empathy, defined as the ability to understand and respond to the emotions of others, is essential for developing meaningful social connections. Adolescents with autism may also experience difficulties interpreting facial expressions, tone of voice, and gestures, which can complicate their understanding of the social norms required in group contexts. Recent studies suggest that development of prosocial behaviors and socio-emotional skills—including communication, conflict resolution, emotional regulation, and emotion recognition—is crucial for successful social functioning among adolescents with autism [9,10].

Compared to their typically developing peers, adolescents with autism are less likely to utilize negotiation or compromise strategies to resolve conflicts [7]. In some cases, adolescents with autism may respond inappropriately to social situations, such as using humor that is not suitable or misunderstanding irony and sarcasm, which complicates their ability to maintain appropriate social relationships and leads to inappropriate social responses [11]. Humor processing is also atypical, with autism being linked to both disengagement and over-involvement with humor [12].

In this context, it is important to emphasize the need to develop these skills to improve the quality of life for adolescents with autism and connect them to more meaningful social relationships.

### 1.3. Late ASD Diagnosis

Previous studies on late autism diagnosis during adolescence have recognized that autistic traits in females often go unnoticed beyond early childhood, potentially contributing to later diagnosis [13]. However, despite growing awareness, research on the broader group of individuals diagnosed later in development remains limited. In many cases, late diagnosis is attributed to higher social-communication abilities, a less pronounced autistic clinical profile, average to high cognitive abilities (IQ > 75), and strong linguistic skills [14,15,16], which may help them camouflage their autism-related challenges. This late-diagnosed subgroup also often exhibits comorbid psychopathological conditions such as somatic strain, depressive disorder, or anxiety [17]. Neurodevelopmental and medical conditions such as Attention Deficit Hyperactivity Disorder (ADHD), epilepsy, sleep disorders, and psychiatric disorders all bring heterogeneity to the clinical presentation and oftentimes mask the autistic symptoms [14].

Research has indicated that late-diagnosed autistic youngsters often use adaptive strategies like masking techniques to lessen the impact of autistic traits on their social and communication skills [16,18,19,20]. Individuals diagnosed later in development (ages 6–18) often exhibit intact or advanced linguistic skills, as the absence of early language delays may allow their social communication differences to remain hidden until adolescence, when social demands becomes more complex [21]. Yet, studies that investigated phenotypical characteristics (cognitive, socio-communicative) of late-diagnosed adolescents did not provide information on specific biological sex differences [22,23].

### 1.4. Evaluating the Social and Communication Abilities of Adolescents with ASD

Assessment of social communication in adolescents with autism typically relies on standardized parent-report questionnaires. The Social Responsiveness Scale–Second Edition (SRS-2; WPS, Torrance, CA, USA) [24] is widely used to quantify autistic traits and social deficits [25,26,27]. The Adaptive Behavior Assessment System–Second Edition (ABAS-II; WPS, Torrance, CA, USA) [28] evaluates adaptive and socialization skills [29,30], while the Child Behavior Checklist (CBCL; ASEBA, Burlington, VT, USA) [31] captures autism symptoms, comorbidities, and internalizing and externalizing difficulties [32,33,34].

Although these parent reports are well established, they are subject to caregivers’ subjective perceptions. Consequently, direct observations of spontaneous peer interactions have gained recognition as a more objective and ecologically valid approach to evaluating social behaviors in autistic youth [18,35]. Observational studies provide unique insights into real-time social functioning, yet current tools and methodologies remain limited.

For example, the Peer Interaction Paradigm (PIP) [36] primarily targets reciprocal communication in early to middle childhood. In contrast, the Autism Peer Interaction Observation Scale–Adolescent version (APIOS-A) includes qualitative aspects of communication style, enabling finer analysis of biological sex-related social patterns. Existing observational studies often focus on narrow behavioral domains—such as stereotypy, discourse features, or reciprocity [1,37]—and are typically conducted in educational settings with small, gender-imbalanced samples [2,38]. Moreover, many have been intervention-based rather than naturalistic [39,40].

Expanding observational approaches, particularly those capturing nuanced, spontaneous peer interactions, is therefore essential for understanding social behavior and biological sex differences among autistic adolescents.

### 1.5. The Current Study

Despite the importance of understanding social interaction challenges and biological sex differences among late-diagnosed autistic adolescents, existing observational tools have not provided a comprehensive assessment of spontaneous peer interactions in this group. To address this gap, the present study adapted the Autism Peer Interaction Observation Scale (APIOS) [6]—originally developed for preschoolers—to be developmentally appropriate for adolescents. The preschool version reliably differentiated between autistic and non-autistic children and showed strong convergent validity with established autism measures such as the SRS-2 and VABS-II.

Using this modified version, the APIOS-Adolescent (APIOS-A), we directly assessed the peer interactions of autistic boys and girls diagnosed during adolescence. Complementary parental evaluations were obtained using the ABAS-II [28] to assess socialization skills, the SRS-2 [24] to measure social and behavioral deficits, and the CBCL [31] to examine internalizing and externalizing characteristics known to affect social-communication functioning in this group.

The first aim of this study was to examine biological sex differences across the APIOS-A, ABAS-II, SRS-2, and CBCL. Based on prior findings, we hypothesized that boys would demonstrate lower quality of directly observed social and communication skills than girls on the APIOS-A. We also expected girls to show fewer social deficits on the SRS-2 and ABAS-II, greater internalizing tendencies, and fewer externalizing behaviors compared to boys.

The second aim of this study was to examine biological sex differences in the associations between adolescents’ observed peer interaction behaviors (APIOS-A) and parental reports of their social functioning and personal characteristics (ABAS-II, SRS-2, CBCL). We expected that higher-quality observed peer interactions would be associated with fewer social deficits on the SRS-2 and with higher social functioning on the ABAS-II for both boys and girls. However, because prior research provides limited evidence regarding biological sex differences in these associations, we were unable to formulate specific hypotheses about whether the strength or direction of these relations would vary by sex. Additionally, we predicted that poorer peer interaction behaviors would relate to more internalizing symptoms among girls and more externalizing symptoms among boys.

## 2. Method

Participants were recruited from an outpatient autism center. Inclusion criteria included adolescents aged 12–18 years who met diagnostic criteria for autism spectrum disorder as assessed by the Autism Diagnostic Observation Schedule, Second Edition (ADOS-2; WPS, Torrance, CA, USA), and demonstrated cognitive functioning within predefined thresholds. To ensure homogeneity across groups, participants were matched according to chronological age and cognitive levels. Recruitment methods took place between June 2021 and July 2022. Parents were contacted and provided with detailed information regarding the study’s objectives and procedures prior to given informed consent for participation.

Participants comprised 61 adolescents aged 12–18 years (31 boys, 30 girls) with cognitive functioning within the average range (IQ ≥ 75) who received a first-time ASD diagnosis within the three years preceding the study at an outpatient autism center. Inclusion criteria were (a) age between 12 and 18 years; (b) meeting ASD diagnostic criteria on the Autism Diagnostic Observation Schedule, Second Edition [41]; and (c) verbal IQ ≥ 75 as measured by the Peabody Picture Vocabulary Test, Fifth Edition, (PPVT-5; Pearson, Bloomington, MN, USA) measuring verbal IQ specifically (PPVT-5) [42]. Participants had a verbal IQ ≥ 75, with scores ranging from 88 to 128. Boys and girls were matched for chronological age, ADOS-2 scores, PPVT-5 scores, and maternal education level.

Maternal education was used as an indicator of socioeconomic status (SES) due to its reliability in reflecting access to resources and its link to developmental and health outcomes [43]. It has also been associated with autism diagnosis rates [44] (see Table 1). Given the limited parental participation in this study, with only 6 fathers compared to 55 mothers, we focused on maternal education as a more robust measure. The primary caregivers of the adolescents included 55 mothers and 6 fathers aged 31–67 years (M = 49, SD = 9), with parental education ranging from 12 to 22 years.

### 2.1. Measures

Study measures included direct observation of social interaction, coded using the observation tool adapted for adolescents, and three questionnaires rated by parents on their adolescent’s social functioning and personal characteristics.

### 2.2. Observation of Adolescents’ Peer Interactions

The Autism Peer Interaction Observation Scale—Adolescents (APIOS-A)—was adapted for this study from the preschooler version (APIOS-Y) [6] to map the core areas of social difficulty or challenge facing boys and girls with autism during adolescence. A comprehensive literature review identified key social difficulties, highlighting the necessity of adapting the APIOS-A to account for developmental and social differences from younger children.

Based on this review, categories considered less relevant for adolescents were removed, and new subcategories were introduced, including discourse, comprehension of direct and indirect humor, and cognitive flexibility. This adaptation involved collaboration with experts from the university and the autism center, providing valuable insights into adolescent behaviors. Following the adaptation, the reliability and validity of the APIOS-A were evaluated using scientific criteria, yielding positive results.

Compared to the preschooler version, APIOS-A expands the assessment of nonverbal communication to include facial expressions and introduces several social interactive behaviors. The discourse category was also expanded to encompass humor and irony, while categories related to social and imaginative play typical of early childhood were removed.

The APIOS-A comprises six general categories related to social communication: nonverbal communication, social behaviors, prosocial behaviors, conversation skills, general interaction quality indicators, and negative interactions. Three categories contain subcategories that identify specific interactive behaviors:

- Nonverbal communication: Eye contact, gestures, and facial expressions.
- Social behaviors: Proximity, social sharing, managing conflicts, cognitive flexibility, and understanding humor/irony.
- General interaction quality indicators: Quality of initiative responses, reactive responses, and enjoyment of interactions.

In total, the APIOS evaluates 14 specific interactive behaviors across these six categories. The internal consistency of the observation tool was assessed using Cronbach’s alpha, resulting in the following scores: Nonverbal Behaviors (α = 0.82), Social Behaviors (α = 0.82), Pro-Social Behaviors (α = 0.81), Discourse Skills (α = 0.80), General Indicators (α = 0.80), and Negative Behaviors (α = 0.82). The findings support the tool’s validity, reliability, sensitivity, and specificity. In addition, expert evaluation demonstrated high inter-rate agreement between two clinical social work experts who evaluated the behaviors of 22 participants. Agreement was assessed using the percentage of identical ratings and Kappa statistics, with results detailed in Table 2.

Peer Interaction Observation Procedure: Adolescents’ peer interactions were observed during two naturalistic group activities conducted in a quiet room at the Marot Center, equipped with two fixed cameras in a closed-circuit setup. Each 30 min session was videotaped for later coding. Activities were conducted in same-sex groups of 3–5 participants, matched by age (within two years) and verbal IQ (within 15 standard score points on the PPVT-5) [42]. All participants met ASD diagnostic criteria on the ADOS-2 [41] with a severity cutoff score of ≥7, though this score was not used for group assignment. Eighteen groups were formed (nine all-boys, nine all-girls). The first activity, a semi-structured conversation, lasted 15 min. Participants sat freely around the central table. The facilitator (a trained social worker) introduced the activity as follows: “In front of you are cards showing different topics related to your life. Each of you is invited to choose one or more cards, introduce yourself, and share your thoughts about the card(s) you picked.” The cards displayed short, illustrated statements such as “Being a good friend is important,” “Succeeding in school matters most,” and “I want to be independent in my life.” The facilitator encouraged equal participation and ensured turn-taking, but avoided guiding the content of discussion, allowing spontaneous interaction to emerge naturally between peers. Conversations varied between facilitator-directed exchanges and reciprocal dialog among participants.

The second activity was a collaborative construction task using Marble Works^®^, where participants jointly built and tested a functional marble maze. The facilitator provided only brief task instructions and minimal intervention, promoting cooperation, shared problem-solving, and peer communication—behaviors shown to elicit authentic social engagement in autistic youth [45]. Both activities concluded with a short collaborative clean-up.

Coding of Videotaped Interactions: A mean score was calculated for each of the APIOS-A, including 6 general categories and 14 specific behaviors in subcategories, where lower scores indicated more adaptive peer interaction on a scale ranging from typical behavior (1) to atypical behavior (4).

To assess interrater reliability, two expert clinical social workers, both with extensive experience in the field of autism, independently coded 36% of the videotaped interactions of the participants (*n* = 22). The coding process was conducted in a blind manner, ensuring that both raters remained independent to maintain objectivity. The agreement between the raters was notably high, ranging from 0.70 to 1.00 for most items on the APIOS-A, with the coefficients being statistically significant at *p* < 0.001 (refer to Table 2). Once the reliability among the coders was established, the first author independently completed the remaining observations, thereby creating a personalized profile that reflects the severity of social and communication difficulties for each adolescent.

### 2.3. Parent-Rated Social and Communicative Behavior

Parents completed three standardized questionnaires assessing adolescents’ social, communicative, and behavioral characteristics.

Social Responsiveness Scale, Second Edition (SRS-2) [24]: The 65-item SRS-2 quantifies ASD-related social impairment and its severity across two DSM-5–aligned dimensions: Social Communication and Interaction (53 items) and Restricted and Repetitive Behaviors and Interests (RRBIs) (12 items). Parents rated each item on a 4-point Likert scale (1 = not true to 4 = almost always true), with lower scores reflecting less severe autistic symptomatology. The SRS-2 demonstrates excellent internal consistency (Cronbach’s α = 0.97) and strong external validity [25].

Adaptive Behavior Assessment System, Second Edition (ABAS-II) [28]: The Socialization subscale (30 items) of the ABAS-II was used to assess adaptive social functioning, including interpersonal skills, responsibility, rule-following, and self-esteem. Items were rated on a 4-point scale (0 = not able to 3 = always/almost always), with higher scores indicating greater adaptive functioning. The ABAS-II shows high reliability (α = 0.95–0.98; test–retest r ≈ 0.87).

Child Behavior Checklist (CBCL) [31]: The 113-item CBCL for ages 12–18 assessed emotional and behavioral problems across two broadband domains: Internalizing Problems and Externalizing Problems. Items were rated on a 3-point scale (0 = not true to 2 = very/often true), with higher scores indicating greater symptom severity. The CBCL demonstrates strong psychometric properties in ASD samples (α = 0.91–0.97) [46].

### 2.4. Procedure

The families recruited for the current study were taken from the pool of all families referred to the Marot Autism Center between 6/2021 until 7/2022. Data collection was conducted from June 2021 to July 2022 according to the following inclusion criteria: (a) age 12–18 years, (b) first-time ASD diagnosis within the past three years, and (c) IQ ≥ 75. Adolescents with comorbid psychiatric, cognitive, or neurological conditions were excluded.

Parents provided written informed consent for participation, and adolescents provided assent prior to data collection. Confidentiality and the right to withdraw at any stage were ensured. Two participants (one male, one female) withdrew voluntarily and were excluded from analyses. This study received approval from the Institutional Ethics Committee of Ichilov Hospital, which was convened in June 2021 and granted written permission for data collection for one year. Due to delays in data collection caused by the COVID-19 pandemic, we requested and were granted an extension of two additional months, extending the data collection period until August 2022.

The procedure comprised three stages. (1) Parent assessment: Primary caregivers completed the SRS-2, ABAS-II, and CBCL questionnaires during an individual interview with the first author. (2) Adolescent assessment: Each adolescent participated in an online meeting during which assent was obtained and the PPVT-5 was administered. Twelve adolescents scoring below 88 on the PPVT-5 were excluded. (3) Observation phase: The remaining 61 adolescents attended a single in-person group session at the Marot Center for video-recorded peer interaction observation. Participants were assigned to same-sex groups of 3–5 members, matched by age, PPVT-5 score, and ADOS-2 calibrated severity score (≥7).

### 2.5. Data Analyses

Analysis of our study data included tests of differences between boys and girls across directly observed peer interaction behaviors (APIOS-A) as well as parent-reported social functioning behaviors (ABAS-II, SRS-2) and internalizing/externalizing behaviors (CBCL) within a group level design. That is, these indicators were measured in 18 small groups of 3–5 participants each (mean group size: 3.4, SD = 0.8). Note that homogeneity of core characteristics (sex, age range within 2 years, ADOS-2 calibrated severity score of ≥7, IQ within one SD) was an allocation criterion to increase within-group similarity and between-group dissimilarity. Correlations between observed interactive behavior (APIOS-A) and social functioning (SRS-2, ABAS-II) and personal characteristics (CBCL internalizing and externalizing behaviors) were assessed in relation to adolescents’ sex, all of which were individual-level indicators.

Preliminary intra-class correlations (ICCs) were calculated to determine between-group weight in the total variance. By design, the inclusion of group-level data ensured that standard errors were not underestimated and that both sources of variation (individual and group) were expressed. In half of the indicators, the ICC was above 0.05, which is a common threshold for inclusion [47]. These values were calculated within an “unconditional” model, namely without predictors; thus, the meaning of the ICC was the potential for group-level indicators to add to the percent variance explained in further modeling.

Despite the group-level variance as expressed in the ICCs, the current study’s comparisons across groups did not show differences. Thus, we expanded the model to include adolescents’ biological sex while controlling for age effect on our study questions, in two steps. In the first step, we tested biological sex differences in response to the first study question. In the second step, we entered an interaction term between biological sex and each of the independent indicators to test whether the associations were inconsistent across boys versus girls. To increase sensitivity to possibly inconsistent time changes across groups, the rejection criterion for the interaction effect was increased to *p* < 0.10, which is still within the common requirements to prevent Type I error [47,48]. We applied a two-level linear regression modeling approach in which the intercept, but not the other effects, was set to vary randomly across groups, namely a random intercept model. For sex differences, we applied two different modeling strategies: (a) test of the biological sex effect as a covariate, and (b) separate tests for boys and girls. All analyses were performed using the SPSS V.26 (IBM Corp., Armonk, NY, USA) [49] and Mplus V.8.3.1 (Muthén & Muthén, Los Angeles, CA, USA) [50] statistical packages.

## 3. Results

The data analysis reveals that boys and girls exhibited subtle differences in behavioral patterns across several measured categories. For instance, boys displayed difficulties in social functioning, while girls showed a tendency for improvement in social performance, as reflected in higher parent ratings.

**Figure 1:**
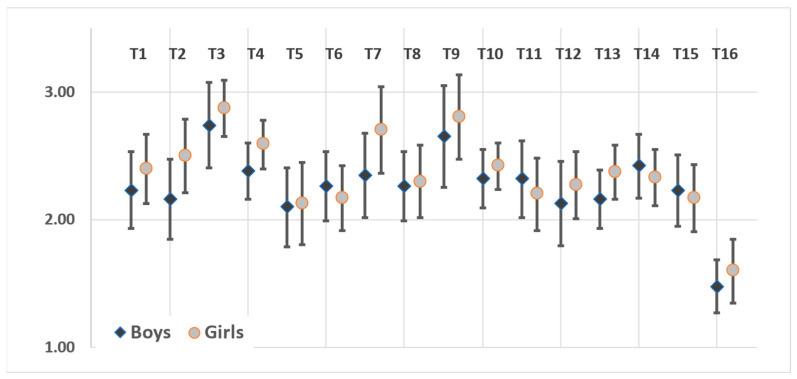
Gender differences across various communication measures.

Observed Peer Interactive Behaviors (APIOS-A): Figure 1 presents boys’ and girls’ peer interaction behaviors (APIOS-A), analyzed using two-level linear regression controlling for chronological age (CA). Contrary to expectations, no significant biological sex differences were found across the six APIOS-A categories or subcategories. Overall, both boys and girls showed social interaction skills within the deficit range (scores ≥ 2).

Parent-Reported Socialization (ABAS-II), Autistic Symptomatology (SRS-2), and Personal Characteristics (CBCL): As shown in Table 3, and consistent with the APIOS-A results, no significant sex differences emerged for socialization (ABAS-II) or internalizing and externalizing behaviors (CBCL). A near-significant difference was observed on the CBCL rule-breaking subscale, with boys showing slightly higher scores than girls (4.26 vs. 3.10, respectively ~ *p* < 0.10). Boys also scored higher on the other problems subscale (6.72 vs. 4.42 respectively, *p* < 0.05). For the SRS-2, a significant sex difference was found only for the behavioral mannerism dimension, with girls exhibiting higher (more severe) RRBIs than boys (0.02~ vs. −0.01~ respectively, *p* < 0.05), while no differences were found in the social communication and interaction domain.

### Biological Sex Differences in Observed Interactive Behaviors’ Associations with Parent-Reported Social Functioning

The findings indicate that boys and girls exhibited different behavioral patterns, especially in the relationships between parent measures and social behaviors. Boys showed social difficulties, while girls tended to improve in social functioning with higher parent ratings.

APIOS-A and ABAS-II Socialization: Table 4 presents the interaction effects between biological sex and ABAS-II Socialization scores on observed peer interactions (APIOS-A). In both the full model and sex-specific analyses, near-significant effects (*p* < 0.10) were found for four APIOS-A behaviors: flexible thinking, social understanding and use of humor/irony, conversational skills, and initiation. Significant negative interaction effects (sex × ABAS-II Socialization) emerged for flexibility (*b* = −0.02, *p* < 0.10), use of humor (*b* = −0.03, *p* < 0.10), and initiation-overall (*b* = −0.02, *p* < 0.05). These effects indicated that higher parent-rated socialization was associated with fewer observed social deficits among boys, whereas for girls, correlations were positive but non-significant. A positive interaction effect for conversational skills (*b* = 0.03, *p* < 0.10) suggested that better socialization related to fewer conversational difficulties, though no significant sex differences emerged in this domain.

In the separate models, boys showed significant negative associations between ABAS-II Socialization and both social understanding/use of humor (*b* = −0.02, *p* < 0.05) and initiation-overall (*b* = −0.02, *p* < 0.01), while these relationships were non-significant among girls, consistent with the combined model.

APIOS-A and SRS-2: Table 4 presents the interaction effects between sex and SRS-2 social impairment ratings on observed peer interactions (APIOS-A). A significant positive interaction (sex × SRS-2) was found for conflict resolution (*b* = 0.05, *p* < 0.05), indicating that higher parent-rated social impairment was associated with greater observed difficulties in managing conflicts. However, when analyzed separately by sexes, this association was not significant for either group. Another significant interaction emerged between sex and the SRS-2 behavioral mannerism dimension on APIOS-A sharing behaviors (*b* = 0.03, *p* < 0.05). Among boys, higher behavioral mannerism scores were linked to greater sharing difficulties, whereas among girls, the relationship was negative—higher behavioral mannerism scores were associated with fewer sharing deficits, though significant negative correlation only in the separate model.

APIOS-A and CBCL Internalizing Behavior: As shown in Table 5, significant sex × CBCL–Internalizing interactions were found for observed eye contact, sharing, and overall social behavior on the APIOS-A. Higher levels of internalizing behaviors were generally associated with greater peer interaction deficits, though the pattern differed by sex.

Among boys, higher internalizing scores were significantly correlated with poorer eye contact and positively, but non-significantly, associated with greater deficits in sharing and overall social behavior. Among girls, the opposite trend emerged: higher internalizing scores were linked to fewer deficits in all three behaviors, with a significant negative correlation only for sharing. Overall, internalizing problems predicted poorer observed peer interactions among boys but tended to relate to better social functioning among girls.

APIOS-A and CBCL Externalizing Behavior: As shown in Table 5, significant sex × CBCL–Externalizing interactions were found for proximity, conflict resolution, flexible thinking, social understanding and use of humor, overall social behavior, and overall responsiveness. Among boys, higher externalizing scores were consistently associated with greater deficits across these APIOS-A behaviors (except for social understanding and humor), indicating poorer peer interaction skills with increased externalizing problems. In contrast, girls showed the opposite pattern: higher externalizing scores were related to fewer observed deficits. These negative associations reached significance for conflict resolution, flexible thinking, social understanding and humor, and overall social behavior.

Taken together, these findings indicate that externalizing behaviors were linked to greater social difficulties among boys but to relatively better social functioning among girls, reflecting possible sex-related differences in how behavioral problems are expressed during peer interactions.

## 4. Discussion

The present study demonstrates the utility of the newly adapted APIOS-A observational tool in profiling peer interactions among late-diagnosed autistic adolescents, capturing both social-communicative challenges and strengths across biological sexes. Overall, no statistically significant sex differences were identified across most dimensions of social-communicative interactions. However, girls demonstrate subtle advantages in cognitive flexibility and in social behaviors such as sharing and emotional understanding. Parental assessments using the SRS-2, ABAS-II, and CBCL revealed a similar pattern, with only minor differences between boys and girls. These findings contribute to a more nuanced understanding of peer interactions among autistic adolescents and may support the development of more targeted and gender-sensitive social interventions.

Based on the previous literature, which has largely relied on parent reports rather than direct observations [25,51], we expected autistic girls to display higher-quality spontaneous peer interactions than boys [9,42,52]. However, this hypothesis was not supported.

The findings indicate that while girls exhibited slightly higher mean scores on most dimensions of the APIOS-A, these differences were not statistically significant. Both sexes demonstrated similarly limited social-communicative abilities, reflecting atypical behaviors. The APIOS-A effectively identifies social challenges common to both sexes, including opportunities for enhancing gesture use and cognitive flexibility, while also highlighting relatively better management of negative behaviors.

This pattern of non-significant sex differences was mirrored in parent ratings on the ABAS-II and SRS-2 (except for behavioral mannerisms). Several explanations may account for this finding. First, the late diagnosis of participants may reflect milder autistic traits or compensatory mechanisms that emerge during adolescence [5]. Second, shared social experiences among late-diagnosed autistic adolescents could produce similar behavioral profiles across sexes. Third, the semi-structured group context of the current study might have reduced opportunities for observing sex-specific behaviors; naturalistic settings such as school recess might elicit clearer sex-related contrasts. Moreover, since all participants were autistic, the absence of typically developing peers may have limited the salience of sex-related social patterns. Research indicates that well-developed social skills can reduce social isolation and improve coping mechanisms for anxiety and depression [26,53]. Gender differences may influence how adolescents with ASD develop social skills, highlighting the need for further research. Social experiences with peers have proven effective in enhancing these skills [39,54].

Finally, socio-emotional development is crucial, as it affects adolescents’ ability to form relationships, understand emotions, and navigate social situations [55,56].

Interestingly, the only significant sex difference on parent reports was found on the SRS-2 Behavioral Mannerisms subscale, where girls exhibited higher levels of repetitive or focused behaviors than boys. This result diverges from most previous findings [57,58] suggesting greater behavioral rigidity in autistic males. However, developmental factors may contribute to this pattern. As Allely et al. [59] noted, behavioral mannerisms in autistic females often differ in content and intensity, which may not be adequately captured by standard instruments. Such under-detection may contribute to delayed identification of autistic girls, emphasizing the need for sex-sensitive assessment tools.

Our findings regarding personal characteristics partially supported the hypothesis that boys exhibit more externalizing behaviors than girls [32,60]. A systematic review and meta-analysis by Wood-Downie et al. [23] highlighted gender differences in social attention and peer relationships, indicating that autistic females demonstrate superior social interaction and communication skills compared to their male counterparts. Furthermore, in adolescents with autism, males were found to have higher scores in Sensory Stimulation and Motor Stereotypes, while females exhibited increased anxiety and affective symptoms over time [58]. Additionally, Carpita et al. [61] explored eating disorders in autistic women and found that camouflaging behaviors significantly contribute to emotional distress, empathy deficits, and a sense of inauthenticity which serve as protective mechanisms against challenges related to social acceptance. Girls with autism employ camouflage strategies to navigate social challenges, such as mimicking the behaviors of their peers or suppressing emotional expressions, which may significantly influence their experiences in social interactions. This underscores the importance of recognizing the distinct social dynamics faced by autistic females, calling for informed interventions that address their specific emotional and social needs.

Boys indeed scored higher on CBCL externalizing problems, particularly rule-breaking and “other” externalizing behaviors, aligning with prior research [62,63]. Conversely, girls showed slightly higher—but non-significant—scores on Internalizing Problems, consistent with broader trends but possibly obscured by sample size or within-group variability.

Overall, the findings highlight the complexity of distinguishing male and female autistic phenotypes during late-diagnosed adolescents. The results underscore the value of combining direct observational methods with parental evaluations to better understand sex-related social functioning and to inform tailored diagnostic and intervention approaches for late-diagnosed autistic adolescents.

We hypothesized that the relationships between adolescents observed interactive behaviors (APIOS-A) and parent-reported measures of social-communicative abilities (ABAS-II, SRS-2) and personal characteristics (CBCL) would show different patterns between boys and girls. Specifically, we aimed to examine whether the nature and strength of these associations varied by sex. This hypothesis was partially confirmed, as several interaction trends were identified.

Correlations between adolescents’ observed peer behaviors and parent-rated socialization revealed several significant links, mainly among boys. For boys only, better understanding and use of humor during peer engagement (APIOS-A) were associated with higher socialization scores on the ABAS-II. Boys’ socialization also showed a near-significant positive correlation with more proficient spontaneous conversation skills during peer interaction. For both sexes, lower socialization was significantly related to reduced social flexibility. Prior studies have linked humor appreciation with greater cognitive flexibility and communication skills [12] and found that boys use humor more frequently and effectively than girls, relating it to better social skills and lower anxiety [64]. Additionally, among boys only, a significant negative correlation emerged between the quality of social initiations (APIOS-A) and socialization (ABAS-II).

Although sex differences in observed behaviors did not reach statistical significance, several near-significant trends emerged, suggesting directions for future study. Specifically, conflict management and disagreement skills (APIOS-A) showed opposite associations with parent-rated social and communication behaviors (SRS-2) for boys and girls: among girls, better conflict management related to fewer social impairments, whereas among boys, greater conflict difficulty corresponded with more severe impairments. These non-significant tendencies align with prior research showing sex differences in conflict resolution—girls report more conflicts and guilt, while boys tend to cope more openly and effectively [9,64], highlighting the need for further exploration of these dynamics.

Another notable sex-specific pattern involved sharing behaviors (APIOS-A) and the SRS-2 behavioral mannerism dimension. For boys, higher behavioral mannerisms were associated with more sharing difficulties, while for girls, greater RRBIs corresponded with better sharing abilities. These findings align with prior reports indicating that autistic boys typically exhibit more over stereotyped behaviors, whereas girls display subtler, compulsive, or sameness-driven patterns [38]. Further research is needed to clarify these contrasting associations and their developmental implications.

Examining associations between observed behaviors (APIOS-A) and parent-rated internalizing and externalizing characteristics (CBCL) revealed several sex-specific patterns. While no significant sex differences were found between overall social behaviors and internalizing scores, distinct trends emerged for specific behaviors. Among boys, higher internalizing scores correlated with poorer eye contact, whereas among girls, higher internalizing symptoms were linked with better social sharing. These opposite directions suggest that internalizing difficulties manifest differently across sexes—limiting social engagement for boys but potentially enhancing prosocial tendencies for girls.

These findings align with prior evidence showing that autistic girls often experience internalizing difficulties and display characteristic camouflaging behaviors, often accompanied by anxiety, depression, and eating disorder symptoms [32,58,62,65]. In contrast, Guerrera et al. [38] reported stronger internalizing problems in autistic boys, who may experience greater social challenges. Such discrepancies could reflect parental bias in reporting or developmental changes in behavior from childhood to adolescence.

Regarding externalizing behaviors, boys’ higher CBCL externalizing scores were significantly correlated with poorer peer interaction abilities across several APIOS-A domains, including managing interpersonal distance, disagreements, conflicts, and cognitive flexibility indicating that greater behavioral problems co-occurred with more impaired social functioning. Conversely, for girls, higher externalizing scores were associated with less impaired social behaviors, such as better conflict management, flexibility, humor, and overall social competence. This pattern, consistent with prior findings that boys show more externalizing problems than girls [32,34,60], suggests that even when girls display externalizing tendencies, they may maintain better social functioning possibly due to camouflaging strategies typical of autistic females [65,66].

## 5. Conclusions

Our findings underscore the need for gender-sensitive assessment tools to understand the challenges faced by late-diagnosed adolescents. While no significant gender differences were found in most social-communicative dimensions, subtle variations in cognitive flexibility and emotional sharing were observed.

Despite the small sample size, our framework provides a holistic view of interactions among adolescents with communication challenges. These results highlight the importance of tailored interventions for improving communication and social skills, addressing individual needs and guiding future research.

### 5.1. Study Implications

This study offers initial insights into the social functioning of adolescents diagnosed with autism during adolescence (ages 12–18), an understudied group. Using a newly adapted observational tool to assess spontaneous peer interactions, alongside parent reports, provided a nuanced view of participants’ social-communicative behaviors.

Although clear sex differences were not found, combining direct observation with parental evaluations highlighted shared and divergent patterns in social functioning. The inclusion of 61 adolescents who received their first diagnosis shortly before participation allowed exploration of factors potentially linked to delayed identification. Prior research suggests that later diagnoses often occur among individuals with milder symptoms and relatively strong cognitive and linguistic abilities [14,15,16,67].

Direct observation during adolescence offered valuable insight into social and communicative behaviors at this complex stage of development. While no consistent sex differences emerged, the observational data contributed to a more refined understanding of the social challenges faced by both autistic boys and girls, informing more tailored intervention goals. The adapted APIOS-A tool may serve practitioners to develop tailored treatment plans based on specific social behaviors of autistic individuals. It identifies areas needing additional support, monitors the impact of interventions on social behaviors over time, and serves as a foundation for creating targeted programs to enhance social skills.

Meaningful associations between APIOS-A metrics and parent-report measures reflected similar patterns of social-communication challenges across sexes. Tentative trends suggested that girls may show slightly better conflict management, disagreement handling, and cognitive flexibility but have more difficulty understanding and using humor. Boys appeared to experience greater challenges in nonverbal behaviors and social sharing, though their conversational and humor-related abilities were somewhat stronger. These nuanced patterns emphasize the need for further research into sex-related differences in social functioning among autistic adolescents.

### 5.2. Study Limitations and Recommendations for Future Research

This study has several limitations. First, participants were matched on age, sex, cognitive level, and autism severity, resulting in homogeneous groups, which may have limited our ability to detect sex differences effectively. Additionally, all participants were diagnosed during adolescence, which could minimize observable differences due to similar symptom severity and normative cognitive functioning.

Secondly, the findings indicate a potential need to expand the categories in our observational tool. This enhancement could help distinguish, measure, and capture differences between boys and girls, as well as other relevant factors. A broader approach could involve exploring common conversation topics among adolescents, such as personal matters and hobbies, while also examining potential gender differences in these discussions. Additionally, distinguishing between direct and indirect communication styles could provide deeper insights into social interactions. Furthermore, documenting emotional responses—like laughter, crying, and anger—during these interactions would help in understanding how participants react to one another.

The observational tool employed in this study has several limitations. Firstly, as noted by Ahn et al. [68], the judgments of the observer may be influenced by subjective biases, which can affect the reliability of the data collected. Furthermore, behaviors were measured in controlled settings, leading to potential discrepancies between the observed behaviors and those exhibited in natural environments [68]. Due to the relatively small sample size, extensive adjustment for multiple potential confounders was avoided in order to reduce the risk of overfitting and unstable parameter estimates. Instead, strict inclusion criteria were used to enhance group homogeneity and reduce potential confounding bias. Nevertheless, residual confounding cannot be ruled out. Lastly, the results may lack external validity, as they might not accurately reflect the actual social behaviors of children outside of the assessment conditions [68]. Future research should address these limitations by comparing adolescents with and without autism in more naturalistic settings, such as those in the schoolyard, in order to better capture spontaneous peer interactions and sex-related differences in social behavior.

Creating the sample size could improve statistical analyses, and utilizing the APIOS-A in mixed groups could enhance generalizability. Additionally, integrating multiple data sources—such as self-reports, parent questionnaires, and educational records—alongside direct observations could provide a more nuanced understanding of social and communicative strengths and challenges across diverse contexts.

Finally, the absence of a typically developing control group raises concerns about potential clinical selection bias, which should be addressed in future studies to ensure a clearer comparison of social behaviors.

In conclusion, future research should consider gender differences, social skills assessment tools, and socio-emotional development to deepen our understanding of social skill development in adolescents with ASD.

### 5.3. Human Ethics and Consent to Participate

This study was approved by the institutional Helsinki Committee of Dana-Dwek Children’s Hospital, Tel Aviv Sourasky Medical Center. Approval no. 0059-21-TLV. Date Approval: 4 July 2021 Written informed consent was obtained from the parents of all participating children, and assent was obtained from the children themselves prior to participation.

Informed consent statement: Informed consent was obtained from all subjects involved in the study and from their parents or legal guardians.

## Figures and Tables

**Table 1:** Means and standard deviations for participants’ characteristics.

Characteristic	Boys *n* = 31		Girls *n* = 30	
	M	SD	M	SD
Chronological age (CA) in months	179.10	23.54	184.97	20.19
Autism Diagnostic Observation Schedule (ADOS-2)	9.35	2.58	9.93	3.81
Peabody Picture Vocabulary Test (PPVT-5)	104.94	10.81	103.30	11.95
Mother’s education	14.77	1.93	14.83	1.62

**Table 2:** Interrater reliability (Kappa, percentage agreement) for Autism Peer Interaction Observation Scale—Adolescence (APIOS-A).

Category	Subcategory	Kappa	% Agreement
1. Nonverbal behaviors	1. Eye contact	0.56 ***	72.7%
	1.1 Gestures	0.92 ***	95.5%
	1.3 Facial expressions	0.85 ***	90.9%
2. Social behaviors	2.2 Social sharing	0.86 ***	90.9%
	2.3 Managing disagreements or conflicts	0.91 ***	95.5%
	2.4 Cognitive flexibility	0.87 ***	90.9%
	2.5 Understanding and using humor and/or irony	0.92 ***	95.5%
3. Prosocial behaviors		1.00 ***	100%
4. Conversation skills		0.74 ***	86.4%
5. General quality indicators		0.84 ***	90.9%
	5.1 Quality of initiative responses	0.82 ***	90.9%
	5.2 Quality of reactive responses	0.83 ***	90.9%
	5.3 Ability to enjoy interaction	1.00 ***	100%
6. Negative behaviors		0.93 ***	95.5%
Overall		0.87 ***	91.9%

*** *p* < 0.001.

**Table 3:** Sex and age differences on parent-reported measures using generalized linear mixed model: main effects and marginal means.

Measure		Main Effects		Baseline Intercept	Marginal Means		Relative Fit Index AIC, AICC
		Sex ^a^ b (SE)	Age b (SE)		Boys M (SE)	Girls M (SE)	
Adaptive Behavior Assessment System (ABAS-II)—Socialization		−4.50 (3.89)	0.10 (0.10)	52.89 * (16.76)	66.51 (3.02)	71.00 (3.11)	500.38, 500.60
Social Responsiveness Scale (SRS-2)	Social communication & interaction	−6.10 (4.04)	0.02 (0.11)	79.17 (14.23)	76.98 (3.10)	83.08 (3.10)	463.51, 463.73
	Behavioral mannerism	−8.02 * (4.10)	−0.01 (0.10)	86.01 * (16.04)	73.31 (3.10)	81.34 (3.20)	491.29, 491.51
Child Behavior Check List (CBCL)	Internalizing	−3.10 (3.22)	−0.02 (0.11)	71.23 (13.10)	64.65 (2.30)	67.3 (2.31)	452.41, 452.63
	Externalizing	−3.16 (3.10)	−0.23 (0.11)	63.71 (11.27)	56.26 (2.05)	59.42 (2.07)	436.27, 436.49
	Rule-breaking	2.10 ~ (1.10)	−0.10 (0.10)	3.55 (3.50)	4.26 (1.03)	3.10 (1.04)	301.39, 301.61
	Other problems	2.29 * (1.20)	0.11 (0.01)	−6.39 (5.10)	6.72 (1.00)	4.42 (1.01)	359.06, 359.27

M = Mean; SE = Standard Error; AICC = Akaike Information Criterion Corrected; ^a^ indicates biological sex biological sex coded as a covariate; b indicates estimated parameters; * *p* < 0.05 indicates statistical significance; ~ *p* < 0.10 indicates a statistical trend.

**Table 4:** Interaction effects on the APIOS-A of sex with parent-rated social functioning (ABAS-II, SRS-2).

Interaction	Interaction Effect on APIOS-A Scale Category/Subcategory	Complete Model			Separate Models	
		Combined M (SE)	Boys M (SE)	Girls M (SE)	Boys M (SE)	Girls M (SE)
Sex × Adaptive Behavior Assessment System (ABAS-II)—Socialization	Flexible thinking	−0.02 ~ (0.01)	−0.01 ~ (0.01)	0.01 (0.01)	−0.01 (0.01)	0.01 (0.01)
	Understanding and using humor and/ irony	−0.03 ~ (0.02)	−0.02 * (0.01)	0.01 (0.01)	−0.02 * (0.01)	0.01 (0.01)
	Conversational skills	0.03 ~ (0.01)	0.01 (0.01)	−0.02 (0.01)	0.01 (0.01)	−0.02 (0.01)
	Quality of Initiation	−0.02 * (0.01)	−0.02 ** (0.01)	0.01 (0.01)	−0.02 ** (0.01)	0.01 (0.01)
Sex × Social Responsiveness Scale (SRS-2)—Social dimension	Problem solving	0.05 * (0.03)	0.03 (0.02)	−0.02 (0.02)	0.03 (0.02)	−0.03 (0.02)
Sex × SRS-2—Mannerism dimension	Social sharing	0.03 * (0.01)	0.02 ~ (0.01)	−0.01 (0.01)	0.02 ~ (0.01)	−0.01 ~ (0.008)

** *p* < 0.01, * *p* < 0.05, ~ *p* < 0.10.

**Table 5:** Interaction effects on the APIOS-A of sex With parent-rated personal characteristics (CBCL).

Interaction	Interaction Effect on APIOS-A Scale Category/Subcategory	Complete Model			Separate Models	
		Combined M (SE)	Boys M (SE)	Girls M (SE)	Boys M (SE)	Girls M (SE)
Sex × Child Behavior Check List (CBCL)—Internalizing	Eye contact	0.04 * (0.02)	0.04 ** (0.01)	−0.01 ~ (0.02)	0.04 ** (0.01)	−0.002 (0.02)
	Sharing	0.05 * (0.02)	0.01 (0.01)	−0.03 * (0.01)	0.01 (0.01)	−0.03 * (0.01)
	Social behavior—average of all subcategories	0.03 ~ (0.02)	0.01 (0.01)	−0.01 (0.01)	0.01 (0.01)	−0.01 (0.01)
Sex × CBCL—Externalizing	Proximity	0.06 * (0.03)	0.03 * (0.015)	−0.03 (0.02)	0.03 * (0.01)	−0.03 (0.02)
	Problem solving	0.08 ** (0.03)	0.03 * (0.015)	−0.04 (0.03)	0.03 * (0.01)	−0.06 * (0.02)
	Flexible thinking	0.06 * (0.02)	0.02 * (0.01)	−0.03 ~ (0.02)	0.02~ (0.01)	−0.04 * (0.02)
	Social understanding and use of humor/irony	0.06 ~ (0.03)	0.01 (0.02)	−0.05 ~ (0.03)	0.01 (0.02)	−0.05 * (0.02)
	Social behavior—average of all subcategories	0.06 ** (0.02)	0.02 ** (0.01)	−0.03 ~ (0.02)	0.02 * (0.01)	−0.03 * (0.01)
	Initiative Responsiveness—average of all subcategories	0.05 * (0.02)	0.04 *** (0.01)	−0.01 (0.02)	0.04 ** (0.01)	−0.01 (0.01)

*** *p* < 0.001, ** *p* < 0.01, * *p* < 0.05, ~ *p* < 0.10.

## Data Availability

The data presented in this study are available on request from the corresponding author.
